# Expression of Dlx-5 and HLX Proteins in Odontogenic Cysts

**DOI:** 10.3390/life15020301

**Published:** 2025-02-14

**Authors:** Sinan Ateş, Uğur Topaloğlu, Mehmet Erdem Akbalik, Şeyma Keleş Karagözoğlu

**Affiliations:** 1 Department of Oral and Maxillofacial Surgery, School of Dentistry, University of Bingöl, 12000 Bingöl, Türkiye; 2 Department of Histology and Embryology, Faculty of Veterinary Medicine, Dicle University, 21280 Diyarbakır, Türkiye; ugur.topaloglu@dicle.edu.tr (U.T.); erdem_akbalik@hotmail.com (M.E.A.); 3 Health Services Vocational School, University of Bingöl, 12000 Bingöl, Türkiye; skeles@bingol.edu.tr

**Keywords:** Dlx-5, HLX, radicular cyst, odontogenic cyst, immunohistochemistry

## Abstract

Odontogenic cysts, commonly detected during routine examinations involving head and neck imaging such as orthopantomograms and computed tomography (CT), are classified into two groups: developmental and inflammatory. Radicular cysts, which belong to the inflammatory group, originate from odontogenic epithelium, while dentigerous cysts of developmental origin are observed as a result of peri-coronal expansion of fluid in the dental follicle. The diagnosis and identification of odontogenic cysts rely on clinical, radiographic, and histological evaluations. This study aimed to demonstrate the expression of Dlx-5 and HLX proteins in radicular and dentigerous cysts. A total of 40 radicular and 40 dentigerous cysts were obtained from patients who visited private oral and dental health clinics in Bingöl and Diyarbakır provinces. After undergoing routine histological procedures, the cysts were stained using Masson’s Trichrome and immunohistochemistry techniques. As a result, the epithelium of radicular cysts was found to be keratinized stratified squamous, with hyaline (Rushton) bodies located within the epithelium. Dentigerous cysts, on the other hand, consisted of non-keratinized stratified squamous epithelium, rete ridges with hyperplastic areas, and inflammatory cell infiltrations. The immunoreactivity induced by Dlx-5 in epithelial and connective tissue cells of radicular and dentigerous cysts was found to be stronger than that of HLX. The positive expression of Dlx-5 and HLX proteins in radicular and dentigerous cysts suggests that these proteins may play a potential role in the pathogenesis of these cysts. Furthermore, it was considered that the expression of Dlx-5 and HLX might help reveal the behavioral differences between odontogenic cysts.

## 1. Introduction

A cyst is a pathological cavity, often filled with fluid, and lined by epithelium. Various developmental cysts have been observed in the head and neck region. Among these, odontogenic cysts are the most common cystic lesions affecting the maxillofacial area. These cysts are classified into two main groups: developmental cysts, including keratocysts and dentigerous cysts, and inflammatory cysts, such as radicular cysts [1,2,3,4]. It has been stated that with some exceptions, epithelial-lined cysts in the bone are observed only in the jaws and are mostly derived from odontogenic epithelium. While the origins of developmental odontogenic cysts are not well understood, inflammatory cysts are known to result from inflammation [5]. Radicular cysts are reported to arise from the prolonged inflammatory process in the bone surrounding the root apex, accompanied by the proliferation of epithelial cells resting the periodontal ligament. On the other hand, dentigerous cysts are developmental in origin and occur due to the peri-coronal expansion of fluid within the dental follicle [6,7,8]. Determining the prevalence of odontogenic cysts, which are widely observed worldwide, is quite challenging. This is because cysts are often asymptomatic and access to imaging techniques is difficult in some areas. However, a retrospective analysis reported that these cysts have a frequency of 13.8% in adults and 11.78% in children [9,10,11]. Odontogenic cysts are usually asymptomatic and can grow to significant sizes before any clinical signs are observed. Therefore, they are often incidentally detected during radiographic examination. As the size of these cysts increases, they may become more clinically apparent, initially presenting as bony hard swellings. With gradual expansion, the bone covering thins, and the lesion extends beyond the bone boundaries, becoming a fluctuating swelling. Slow expansion of the cyst often displaces anatomical structures such as the inferior alveolar nerve in the mandible, but sensory changes are uncommon. When altered sensation occurs, it is often indicative of infection or more aggressive pathology [8,12]. Odontogenic cysts exhibit significant variability in size and histological features. Diagnosis typically involves macroscopic dissection followed by histological tissue preparation to best assess the epithelial lining of the cyst wall. Accurate diagnosis requires a detailed evaluation of radiological images, including the cyst’s location, size, outline, multilocularity, radiolucency/radiopacity, and its relationship with nearby teeth and structures [11,13,14]. Nevertheless, odontogenic cysts are usually detected during routine examinations that include orthopantomograms, such as head and neck imaging. Traditional radiographic techniques (panoramic, occlusal, and periapical radiographs) allow localization of lesions but are not specific [15]. Besides this, for the evaluation and differential diagnosis of maxillary and mandibular lesions, cone beam computed tomography (CBCT) and Magnetic Resonance Imaging (MRI) can be used in addition to conventional panoramic radiography [16]. Among these, CBCT is a notable method as it provides correct information about the location, borders, and effects of the lesions on surrounding structures, displaying them in three dimensions. However, when compared to MRI, it has some disadvantages, the most significant being exposure to X-ray radiation [17]. MRI, on the other hand, provides information beyond image quality, does not expose the patient to the harmful effects of ionizing radiation, and thus helps establish a precise spatial and anatomical diagnosis for treatment. Another advantage of MRI, compared to other imaging methods, is its ability to determine the content of the lesions [18,19].

Homeobox proteins play critical roles in tooth morphogenesis and development. Mutations in homeobox proteins are linked to developmental disorders such as odontogenic lesions [20]. Dlx5, a member of the homeobox protein family, plays a vital role in regulating various biological activities during organogenesis and craniofacial bone development [21]. It has also been noted that DLX5/Dlx5 influences the formation and development of teeth and odontogenic lesions [20,22]. Another homeobox protein family member, HLX/Hlx, is crucial for functions such as visceral organogenesis and the development of the enteric nervous system. Mutations in HLX have been reported to negatively impact the enteric nervous system, causing developmental defects and embryonic lethality [23,24]. HLX has also been observed to be expressed in the mandibular bone arch during embryonic development, suggesting a role in mandibular formation [25]. Furthermore, the H2.0-like homeobox gene (HLX) is thought to play a significant role in normal hematopoietic and stem cell proliferation, differentiation, cancer development, and normal tissue maintenance [26]. The diagnosis, progression, pathogenesis, and recurrence rates of jaw cysts remain controversial due to the numerous cyst-like lesions that can occur in the jaw. Thus, careful diagnosis and treatment of jaw cysts and lesions are necessary [13]. While the diagnosis of cysts may partially rely on clinical examination (e.g., cortical bone expansion, tooth mobility, tooth displacement, pain due to infection), definitive diagnosis requires radiological imaging and histopathological findings [14,27].

The present study aims to (i) determine the expression of Dlx-5 and HLX proteins in radicular and dentigerous cysts, (ii) assess whether the expression intensity differs between the cyst types and (iii) evaluate the potential of these proteins as auxiliary markers for cyst diagnosis using immunohistochemistry.

## 2. Materials and Methods

### 2.1. Patient Characteristics and Collection of Cyst Samples

Ethical approval for this study was obtained from the Non-Interventional Research Ethics Committee of Fırat University with approval number 28370 (10 October 2024). A total of 80 cyst samples, including 40 radicular cysts and 40 dentigerous cysts, were used in the study. The cysts were obtained from patients who visited private oral and dental health clinics in the provinces of Bingöl and Diyarbakır. A preliminary diagnosis of the cysts was made based on the clinical and radiographic findings of the patients. The samples were selected to include equal numbers for each gender and cyst type. All patients were informed about the study, and their consent for the use of their cyst samples was obtained. Patients with non-epithelial cysts or tumors, pregnancy, any treatment technique (surgical excision, radiotherapy), and different systemic infections were excluded from the study. For the histological diagnosis of radicular and dentigerous cysts, the World Health Organization (WHO) Classification of Head and Neck Tumors (2017) was used as a reference [9].

### 2.2. Routine Histological Tissue Processing and Staining

Tissue samples obtained from patients were fixed in a 10% neutral formalin solution for 24 h. Following fixation, the tissues were washed under running tap water for one day. Subsequently, tissue dehydration was performed by passing the samples through a graded alcohol series of 70%, 80%, 96%, and 100%. To clear and harden the tissues, they were sequentially immersed in methyl benzoate for 36 h and in benzene for 90 min. The tissues were then treated in paraffin melted in benzene at 58 °C for 30 min in an incubator and subsequently placed in clean, molten paraffin at 58 °C in an oven for 4 h. Following this process, the tissues were embedded in liquid paraffin to form blocks. Serial sections, 5 μm thick, were obtained from these blocks using a rotary microtome (Leica RM-2125, Leica Biosystems, New York, NY, USA). Some of the sections were placed on standard slides for triple staining, while others were placed on adhesive slides for immunohistochemistry staining. The sections designated for triple staining were first deparaffinized in xylene and then rehydrated through a graded alcohol series of 100%, 96%, 80%, and 70%, with each step lasting three minutes. Subsequently, the sections were stained for morphological evaluation using Masson’s Trichrome staining technique [28].

### 2.3. Immunohistochemical Staining

Serial sections placed on adhesive slides for immunohistochemical staining were subjected to the indirect streptavidin–biotin complex method (Topaloğlu). The sections were deparaffinized by immersing them in xylene I (5 min) and xylene II (5 min). Subsequently, they were rehydrated by passing through a graded alcohol series of 100%, 96%, 80%, and 70%, each for 3 min, followed by washing in distilled water. To block endogenous peroxidase activity in the tissue samples, the sections were incubated in a 3% H_2_O_2_ solution prepared in methanol for 20 min. Next, the sections were washed three times in 0.01 M phosphate-buffered saline (PBS), each wash lasting 5 min. For antigen retrieval, a citrate buffer (0.01 M, pH 6) was prepared, and the sections were incubated in this buffer solution at 95 °C for 20 min, followed by cooling. After cooling, the sections were washed in 0.01M PBS. To block nonspecific staining in the tissues, the sections were treated at room temperature for 15 min with a protein-blocking solution (Ultra V Block, Thermo Fisher Scientific, Lab Vision Corporation). Normal sections were then incubated overnight at +4 °C with Dlx-5 (Rabbit polyclonal, catalog no: STJ9272, St John’s Laboratory) and HLX (Rabbit polyclonal, catalog no: PA5-44857, Invitrogen) primary antibodies diluted at 1/100. Negative control preparations were incubated with PBS under the same conditions. After the reaction, the preparations were washed three times in 0.01M PBS, each wash lasting 5 min. The sections were then incubated at room temperature for 20 min with a biotinylated secondary antibody (Histostain Plus Bulk Kit, Zymed) to facilitate binding to the primary antibody. Following this, the preparations were washed three times in 0.01 M PBS for 5 min each and subsequently incubated for 20 min in a streptavidin–peroxidase solution (Histostain Plus Bulk Kit, Zymed). Afterward, the preparations were washed three times in 0.01 M PBS, each wash lasting 5 min. To demonstrate the antigen–antibody reaction, the preparations were incubated with 3′3-diaminobenzidine hydrochloride (DAB) for 5–15 min, depending on the intensity of the immunoreaction. The reaction was stopped by transferring the preparations into distilled water. For counterstaining, the preparations were immersed in Mayer’s hematoxylin for 2–3 min, followed by washing under running tap water for 4 min. The preparations were then transferred to distilled water, passed through alcohol and xylene series, and mounted with Entellan. Finally, the presence of immunoreactivity in the preparations was observed using a Nikon Eclipse E400 research microscope (Nikon, Tokyo, Japan) equipped with a digital camera (Nikon Coolpix 4500).

### 2.4. SemiQuantitative Evaluation

The findings obtained in the study were assessed based on the intensity score of staining performed using the immunohistochemistry technique. Thus, the positive reactions formed by DLX-5 and HLX in cyst cells were quantitatively evaluated. Immunoreactivity observed in the cells was classified into four levels based on staining intensity: negative (−), weak (+), moderate (++), or strong (+++) [29]. The evaluations were performed by expert researchers (U.T. and M.E.A.), and average scores were determined. For scoring, five random fields were selected for each cyst section. The localization and expression of DLX-5 and HLX proteins in the selected areas were examined at magnifications of ×40, ×100, and ×400. As a result, each cyst’s epithelial and connective tissue cells were evaluated (Table 1).

## 3. Results

### 3.1. Patient Details

The number and gender distribution of the cyst types used in the study were equally balanced. For both types, the cysts were predominantly observed in the mandible, particularly in the posterior region of the mandible. The majority of the observed cysts were removed using the enucleation method (Table 2).

### 3.2. Macroscopic and Radiographic Findings

**Figure 1:**
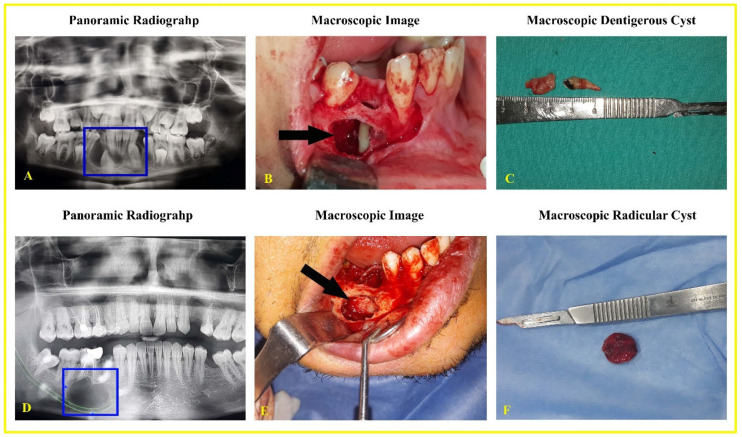
Panoramic radiography and macroscopic images of patients with odontogenic cysts. A Dentigerous cyst (**A**–**C**), radicular cyst (**D**–**F**). Cystic areas are shown in panoramic radiography (blue square) and macroscopic images (arrow).

The radiographic and macroscopic images of the cysts are shown in Figure 1. Most patients were asymptomatic, and the cysts were detected during routine dental examinations. However, in some cases, symptoms were observed due to infection of the cyst causing pain, or because the cyst led to bone expansion or dehiscence in the affected area. Dentigerous cysts were identified as predominantly unilocular radiolucent areas with radiopaque margins associated with the crown of an impacted tooth (Figure 1A). Radicular cysts, on the other hand, were generally observed as radiolucent areas with well-defined radiopaque margins associated with the roots of teeth (Figure 1D). In surgically enucleated cysts, the cyst epithelium, capsule, and cyst fluid were visible. Enucleated cysts could be removed either as a single piece without losing integrity or in fragments after aspirating the cyst fluid. Once the cyst was enucleated, the site in the bone appeared as a void (Figure 1B,C,E,F).

### 3.3. Histopathological and Immunohistochemical Findings

**Figure 2:**
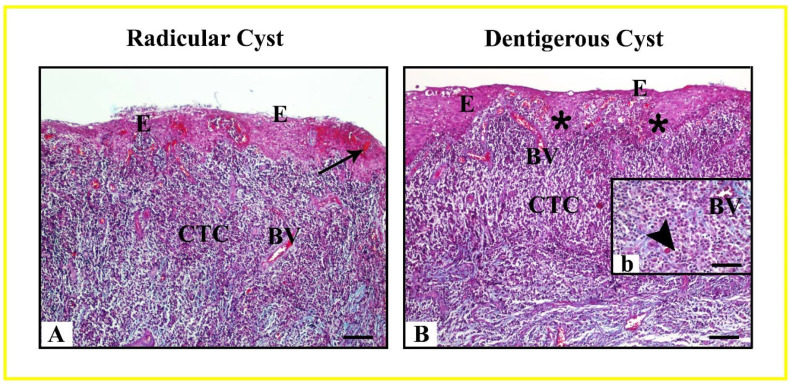
Radicular (**A**) and dentigerous (**B**) cysts are shown with Masson’s Trichrome staining technique; Radicular Cyst: Epithelium (E), Rushton (hyaline) bodies (arrow), Connective Tissue Cells (CTC), Blood Vessel (BV); Dentigerous Cyst: Hyperplastic non-keratinized epithelium (E) with rete ridges (asterisk), Connective Tissue Cells (CTC), Blood Vessel (BV), Giant cell (arrowhead). Scale Bar: 50 μm (**A**,**B**), 25 μm (**b**).

Radicular and dentigerous cysts are histopathologically depicted in Figure 2. The wall of the radicular cyst is lined with a stratified non-keratinized squamous epithelium with varying numbers of layers. As a result of inflammation in the cyst, hyperplasia occurred in the epithelial layer, which exhibited a characteristic arch-like appearance. Additionally, hyaline (Rushton) bodies were observed within the epithelium. Beneath the epithelium, connective tissue cells, collagen fibers, numerous blood vessels, and infiltrations of inflammatory cells were observed (Figure 2A).

Dentigerous cysts, similar to radicular cysts, were found to have a stratified non-keratinized squamous epithelial layer of varying thickness lining the cyst wall. Due to inflammation in dentigerous cysts, rete ridges extensions of the epithelium into the connective tissue were observed, and these regions exhibited hyperplasia. Like radicular cysts, the underlying connective tissue showed connective tissue cells, collagen fibers, inflammatory cell infiltrations, and giant cells (Figure 2B).

**Figure 3:**
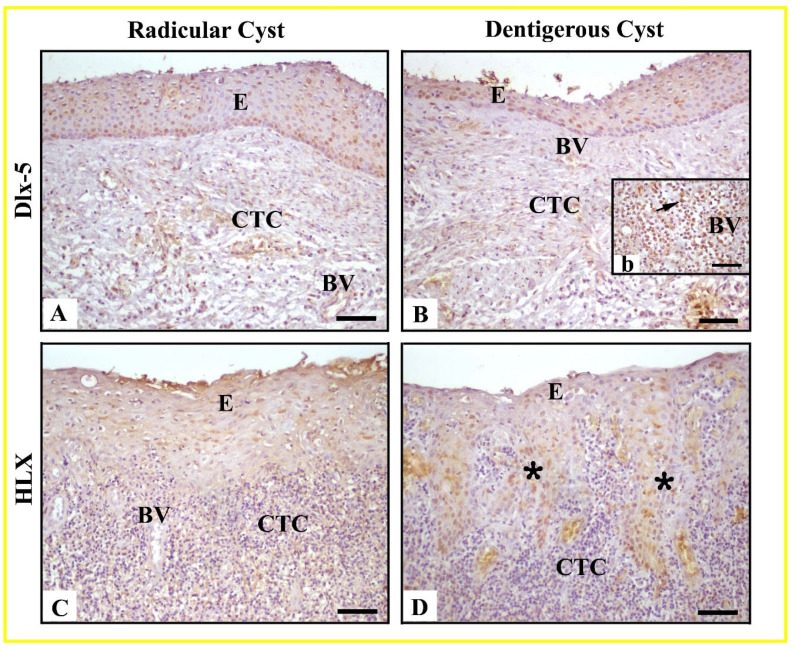
Immunolocalization of Dlx-5 and HLX in radicular (**A**,**C**) and dentigerous cysts (**B**,**D**); Radicular Cyst: Epithelium (E), Connective Tissue Cells (CTC), Blood Vessel (BV); Dentigerous Cyst: Hyperplastic non-keratinized epithelium (E) with rete ridges (asterisk), Connective Tissue Cells (CTC), Blood Vessel (BV), Giant cell (arrow). Scale Bar: 50 μm (**A**–**D**), 25 μm (**b**).

The immunohistochemical expression of Dlx-5 and HLX proteins in radicular and dentigerous cysts is shown in Figure 3. Expression: Dlx-5 showed moderate to strong immunoreactivity in the nuclei and cytoplasm of epithelial cells in radicular cysts. Hyperplastic epithelium exhibited similar staining intensity. Additionally, positive immunoreactivity was observed in some cells within the connective tissue and vascular endothelium (Figure 3A). In dentigerous cysts, the intensity of Dlx-5 immunoreactivity in the epithelium was similar to that in radicular cysts; however, stronger levels were observed in some connective tissue cells, especially those involved in inflammatory cell infiltrations and connective tissue giant cells (Figure 3B).

Expression: HLX exhibited weak to moderate levels of immunoreactivity in epithelial cells of radicular cysts. In dentigerous cysts, immunoreactivity intensity increased from weak to moderate levels in the epithelium, and this intensity was further elevated in rete ridges formed due to epithelial hyperplasia. HLX showed weak immunoreactivity in some connective tissue cells in both radicular and dentigerous cysts, whereas immunoreactivity in blood vessels was found to be negative (Figure 3C,D). To validate the accuracy of Dlx-5 and HLX antibodies, negative and positive controls were utilized. In negative controls, PBS was applied to the sections instead of the antibodies, followed by the same immunohistochemical procedures.

**Figure 4:**
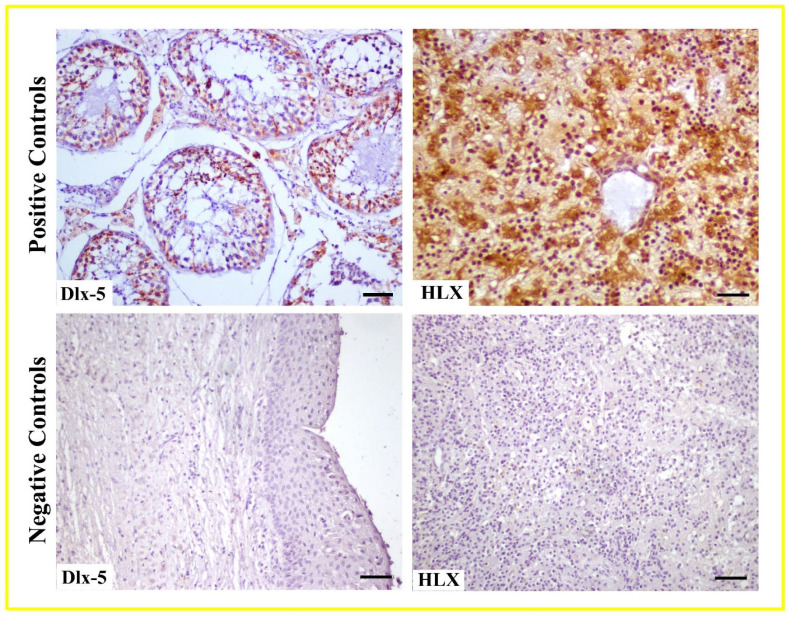
Negative and positive control for Dlx-5 and HLX antibodies. Scale Bar: 50 μm.

Positive controls included testicular tissue for Dlx-5 and liver tissue for HLX (Figure 4).

## 4. Discussion

Odontogenic cysts are typically asymptomatic and are often detected during routine dental radiographic examinations. Recent technological advancements in radiographic devices have significantly enhanced the differentiation and understanding of odontogenic cysts [30]. However, while routine histopathological methods are used for differential diagnosis, the epithelial structure may be disrupted due to the expansion of cystic spaces or inflammatory changes. In such cases, traditional histopathological stains like Hematoxylin-Eosin may be insufficient. Therefore, specific immunohistochemical antibodies have been employed to significantly contribute to the diagnosis and treatment of these cysts [31,32]. In this study, radicular and dentigerous cysts were analyzed clinically, morphologically, radiographically, and immunohistochemically. Previous studies have indicated that odontogenic cysts share histopathological similarities [11]. In our study, we observed histopathological similarities between radicular and dentigerous cysts, highlighting the potential of immunohistochemical analysis for differential diagnosis. Additionally, the expression intensity of proteins used in immunohistochemistry may vary due to factors such as inflammation [11,33]. Consequently, the immunohistochemistry technique is believed to offer novel diagnostic and therapeutic approaches for radicular and dentigerous cysts.

The DLX/Dlx protein family plays critical roles during embryonic morphogenesis and postnatal development. These proteins are involved in the morphogenesis of teeth, eyes, ears, and bones, with their deficiencies or abnormal expressions leading to anomalies. Dlx5 deficiency has been associated with malformations in craniofacial bones, the mandible, and extremities [34,35]. The DLX family has been reported to play essential roles in early odontogenesis, particularly in dental alignment modeling and enamel formation. Dlx5 is expressed in the molar regions of mandibular protrusions [36]. Inactivation of Dlx5 during dental histogenesis in mice disrupts signals regulating ameloblast differentiation, enamel formation, and mineralization, emphasizing the critical role of the Dlx family in tooth development and differentiation [22]. In humans, Dlx5 has been expressed in ameloblastoma, the second most common odontogenic tumor [20]. Furthermore, genetic studies have shown that Dlx5 is involved in the pathogenesis of ovarian cancer, lung cancer, and T-cell lymphoma. It has also been expressed in oral squamous cell carcinoma (OSCC) tissues and cell lines, regulating OSCC, and suggesting its potential as a diagnostic and therapeutic marker [37]. Research indicates that Dlx5 induces tumor formation and development in various cancers, including lung, uterine, and ovarian tumors, by promoting cell proliferation, differentiation, and apoptosis, as well as facilitating metastasis [35,38,39]. Dlx5 has also been linked to metastasis in breast tumors [40]. In our study, Dlx5 showed positive immunoreactivity in both radicular and dentigerous cysts, predominantly in the epithelial layers, especially hyperplastic regions. The immunoreactivity intensity differed in connective tissue cells, with weaker expression in radicular cysts but stronger levels in dentigerous cysts, particularly in connective tissue giant cells and inflammatory cell infiltrations. These findings suggest that Dlx5 may play a role in cell differentiation, division, and proliferation within the cyst wall. Differences in immunoreactivity intensity in connective tissue cells may aid in distinguishing cyst types. Consistent with [37], our results suggest that Dlx5 could serve as a distinctive marker for diagnosing and treating odontogenic cysts, as well as influencing inflammatory responses in dentigerous cysts.

The H2.0-like homeobox gene (HLX) encodes transcription factors involved in tumor formation and plays a critical role in cancer development [41]. HLX has been implicated in various processes, including cell proliferation, differentiation, and maturation. It also induces interferon-γ production and contributes to the formation of T-helper Th-2 cells with Th1 cell functions [42,43]. Abnormal HLX expression has been linked to autoimmune diseases like Graves’ disease and cancers, including gastric and colon cancers, as well as hematopoietic malignancies such as leukemia [26,44]. In colorectal cancer (CRC), HLX acts as a tumor suppressor in the early stages of carcinogenesis but promotes cancer progression in later stages [45]. HLX has also been implicated in acute myeloid leukemia as a promising prognostic and therapeutic target [46]. While studies have primarily focused on HLX’s role in tumor formation, its presence in odontogenic cysts remains unexplored. In this study, HLX exhibited weak to moderate immunoreactivity in the epithelial cells of radicular and dentigerous cysts. Dentigerous cysts showed increased immunoreactivity in rete ridges formed due to epithelial hyperplasia, suggesting HLX’s role in epithelial hyperplasia and its potential as a marker for distinguishing dentigerous cysts.

Homeobox proteins (HMGs) function by binding to DNA and producing proteins that regulate the expression of downstream genes. These proteins exert their regulatory roles through specific targets involved in processes such as organogenesis, cellular differentiation, adhesion, migration, cell cycle, and apoptosis. Disruptions in the regulation of HMGs can lead to dental abnormalities and developmental issues [47], resulting in structural defects and even pathologies such as cysts caused by fluid accumulation. Odontogenic cysts are generally determined as a consequence of the inflammatory process. It is known that humoral and cellular immune responses are effective in the pathogenesis of these lesions [48,49,50], and Hox proteins also play an important role in regulating immune responses. Similar to the odontogenesis process, signaling interactions between various molecules are suggested to play a role in the development of odontogenic lesions [20,51]. Therefore, it is thought that Dlx-5 and HLX, which are sub-members of the Homeobox family, may be involved in the pathogenesis of odontogenic lesions due to disruptions in cellular organization and tissue differentiation resulting from their interactions and changes in expression. Consequently, further studies are needed to investigate the mechanisms underlying odontogenic cyst development.

## 5. Conclusions

The use of Dlx-5 and HLX proteins in cysts diagnosed through radiological imaging methods was found to potentially enhance diagnostic accuracy through observed immunoreactivity. Consequently, it is hypothesized that the detection of Dlx-5 and HLX expression in odontogenic cysts may exert a positive influence on treatment planning and prognostic predictions. Dlx-5 showed higher expression levels compared to HLX, suggesting its greater potential as a distinguishing marker. However, further studies are needed to fully understand the therapeutic functions of these proteins in odontogenic cysts. Incorporating Dlx-5 and HLX expression analyses could enhance diagnostic accuracy, guide treatment decisions, and improve prognostic evaluations for patients with odontogenic cysts.

## Figures and Tables

**Table 1:** Characteristics of patients with radicular and dentigerous cysts.

Parameters		
**Age, mean (min–max)**	Radicular cyst	34.20 (26–69)
	Dentigerous cyst	42.00 (32–63)
**Gender, n (%)**	Male	40 (50%)
	Female	40 (50%)
**Positions on the jaw, n (%)**	Maxillary	20 (25%)
	Mandible	60 (75%)
**Surgery**	Marsupialization	20 (12.50%)
	Enucleation	80 (87.50%)

**Table 2:** Intensity of immunoreactivity for Dlx-5 and HLX in the radicular and dentigerous cyst.

Cells	Dlx-5		HLX	
	**Radicular**	**Dentigerous**	**Radicular**	**Dentigerous**
**Epithelial**	**++/+++**	**++/+++**	**+/++**	**+/++**
**Connective tissue**	**+**	**++/+++**	**+**	**+**

Weak (+), moderate (++) or strong (+++) according to the staining intensity.

## Data Availability

Data are available on request.
